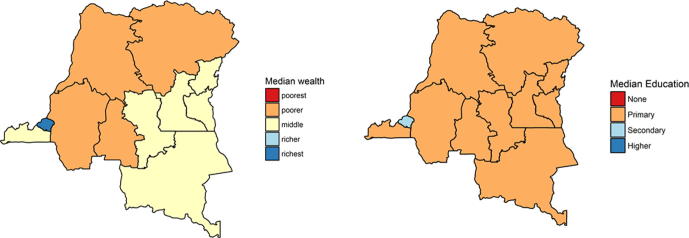# Polio immunity and the impact of mass immunization campaigns in the Democratic Republic of the Congo

**DOI:** 10.1016/j.vaccine.2017.08.063

**Published:** 2017-10-09

**Authors:** Arend Voorman, Nicole A. Hoff, Reena H. Doshi, Vivian Alfonso, Patrick Mukadi, Jean-Jacques Muyembe-Tamfum, Emile Okitolonda Wemakoy, Ado Bwaka, William Weldon, Sue Gerber, Anne W. Rimoin

**Affiliations:** a The Bill and Melinda Gates Foundation, Seattle 98109, USA; b Department of Epidemiology, University of California, Los Angeles 90095, USA; c National Institute for Biomedical Research (INRB), Kinshasa, The Democratic Republic of the Congo; d Kinshasa School of Public Health, Kinshasa, The Democratic Republic of the Congo; e Expanded Programme on Immunization, McKing Consulting, Kinshasa, The Democratic Republic of the Congo; f Division of Viral Diseases, Centers for Disease Control and Prevention, Atlanta 30329, USA

**Keywords:** Poliomyelitis, Immunization, Democratic Republic of the Congo, Seroprevalence, Mass vaccination

## Abstract

**Background:**

In order to prevent outbreaks from wild and vaccine-derived poliovirus, maintenance of population immunity in non-endemic countries is critical.

**Methods:**

We estimated population seroprevalence using dried blood spots collected from 4893 children 6–59 months olds in the 2013–2014 Demographic and Health Survey in the Democratic Republic of the Congo (DRC).

**Results:**

Population immunity was 81%, 90%, and 70% for poliovirus types 1, 2, and 3, respectively. Among 6–59-month-old children, 78% reported at least one dose of polio in routine immunization, while only 15% had three doses documented on vaccination cards. All children in the study had been eligible for at least two trivalent oral polio vaccine campaigns at the time of enrollment; additional immunization campaigns seroconverted 5.0%, 14%, and 5.5% of non-immune children per-campaign for types 1, 2, and 3, respectively, averaged over relevant campaigns for each serotype.

**Conclusions:**

Overall polio immunity was high at the time of the study, though pockets of low immunity cannot be ruled out. The DRC still relies on supplementary immunization campaigns, and this report stresses the importance of the quality and coverage of those campaigns over their quantity, as well as the importance of routine immunization.

## 1 Introduction

Poliomyelitis (polio) is an infectious disease caused by the poliovirus. Like other enteroviruses, poliovirus is transmitted primarily by the fecal-oral route. Poliomyelitis can affect individuals of any age, but primarily involves children aged less than five years. In < 1% of those infected, the virus invades the central nervous system and can cause muscle weakness and acute flaccid paralysis (AFP), usually in the lower limbs, though occasionally progressing to breathing difficulty, and even death [1]. At the time of writing, wild poliovirus (WPV) type 1 continues to circulate in Nigeria, Pakistan, and Afghanistan. WPV type 2 was last seen in 1999, while WPV type 3 was last seen in 2012 [2].

Poliomyelitis is preventable using injectable inactivated polio vaccines (IPV) and live attenuated oral polio vaccines (OPVs) [1], [3]. For the Global Polio Eradication Initiative (GPEI), whose mission is complete eradication and containment of all wild and vaccine-related polioviruses, OPV is the vaccine of choice due to its low cost, ease of delivery, and improved ability to prevent person-to-person transmission [1]. Prior its global withdrawal in April 2016, trivalent OPV (tOPV) was the most commonly used vaccine, providing protection against all three serotypes of poliovirus [1]. Bivalent and monovalent formulations were also used in response to prevalent strains of circulating poliovirus. In April 2016, type 2 containing tOPV was removed from use globally in order to prevent rare adverse events associated with its use, including vaccine-associated paralytic poliomyelitis, and emergence of circulating vaccine-derived poliovirus type 2 (cVDPV2) [4].

**Fig. 1:**
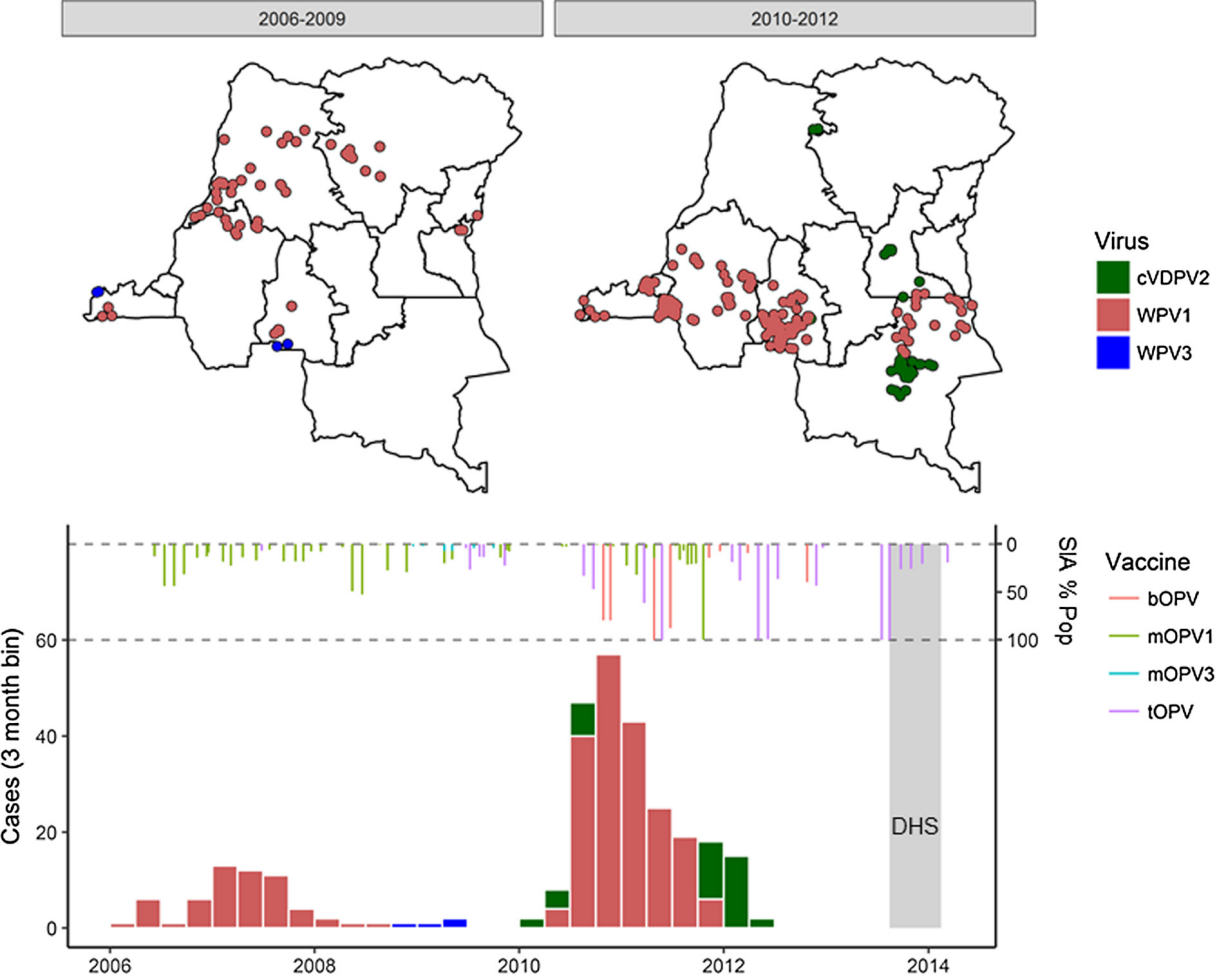
Epidemiology and polio vaccination program history in the DRC. Top panel: the geographic distribution of cases in the periods 2006–2009 and 2010–2012; points are placed randomly in the district where a child was present two weeks prior to the onset of paralysis. Bottom panel: polio AFP case count by three-month bins; above the case counts are bars representing supplemental immunization activities. The grey band shows the period in which the DHS survey was conducted.

In the DRC, the Expanded Program on Immunization (EPI) was introduced in 1978, with the childhood vaccination schedule for polio including four doses of tOPV at birth, 6, 10 and 14 weeks of age. The DRC’s Polio Eradication Program led by the country’s EPI started in 1996, providing additional doses of OPV through house-to-house supplemental immunization activities (SIAs) in areas of the country with a high burden of disease. Until 2001, DRC was endemic for WPV transmission, and was considered a reservoir and exporter of virus to other countries. From 2001 to 2005, no WPV cases were reported in the DRC, and the interruption of WPV transmission was assumed. However, between 2006 and 2011, outbreaks of WPV1 and WPV3 were reported in 10 of 11 provinces as a result of numerous importations from Angola (Fig. 1). In addition, over 10 independent emergences of cVDPV2 were documented during 2004–2012 [5]. The last confirmed WPV case reported in Maniema province with an onset of 20 December 2011. More recently, two cVDPV2 outbreaks were declared in 2016, originating in Maniema and Haut Lomami (formerly Katanga) provinces.

Given the historical importation of poliovirus into DRC and frequent VDPV emergences, achieving and maintaining high population immunity is critical to the success of the GPEI. While immunization campaign coverage monitoring provides operational oversight of individual activities, it gives limited information about their cumulative effect. Vaccination history, when collected, may be highly biased due to imperfect and variable recording and recall [6], [7]. Additionally, even if vaccination history could be obtained, estimates of protective efficacy from polio vaccination vary widely, for instance, between 30 and 100% for 3 doses of tOPV [8]. Lastly, secondary spread of vaccine viruses to contacts of vaccine recipients contributes to population immunity, but varies with population characteristics and is therefore difficult to account for in the absence of immunological data [9]. Therefore, in order to measure the effectiveness of polio immunization activities and identify populations with sub-optimal immunity, serologic assessment of the population is critical.

Nationally representative serologic studies of polio immunity have not been conducted in the DRC, or in the African region more broadly. There have been some targeted assessments of polio immunity, including recent studies in northern Nigeria, India, western China, and Pakistan [10], [11], [12], [13], [14]. In addition, there was a large-scale polio serosurvey in the United States from 2009 to 2010 [15], and a 2013 nutrition survey in Afghanistan which included polio serology [16]. In the DRC, Alleman et al. studied seroprevalence among adult women prior to an outbreak of WPV1 in 2010 and 2011, using samples obtained from ante-natal clinics in the DRC [17]. The authors identified relatively low immunity to type 1 poliovirus among adult women in Kinshasa and Bandundu where the outbreak affected adults, and relatively high immunity in Kasai-Oriental, where the WPV1 cases were exclusively children.

## 2 Data and methods

### 2.1 Demographic and health survey

The second Demographic and Health Survey (DHS) was conducted in the DRC from August 2013 to February 2014. The household survey used a multi-stage stratified cluster design, sampling 18,171 households among 526 clusters and 66 strata, to generate population health and social characteristics representative at the national level, for each of the 11 provinces, and for rural and urban populations [18], [19]. Half of all households were selected for interviews of the adult men, and among these households children 6 to 59 months were eligible for the serological survey.

Data obtained from children included, but was not limited to, demographics, anthropometric measures, health outcomes and vaccination history. After parental consent, dried blood spots (DBS) were collected by heel or finger prick from participating children. Ethical approval was obtained at UCLA Fielding School of Public Health, the Kinshasa School of Public Health and the Centers for Disease Control and Prevention.

### 2.2 Laboratory analysis

Testing for neutralizing antibodies against polio types 1, 2 and 3 was conducted at the US Centers for Disease Control and Prevention [20]. We used the modified poliovirus microneutralization assay which, like the gold standard serum neutralization assay, measures the ability of antibodies in serum or eluted from dried blood spot (DBS) punches to block the infectivity of poliovirus in an in vitro cell culture system. Following collection and in-country processing, the DBS had been stored and shipped at −20 °C with desiccant. On receipt at the laboratory the specimens were logged and randomized. Two six mm punches, equivalent to approximately 6 µl of sera, were collected from each card and processed for the low-volume polio neutralization assay. A series of dilutions of dried blood spot eluate were combined with a fixed amount of virus prior to inoculation of poliovirus-susceptible cells. After five days incubation, a luminescent cell viability reagent was added to detect live cells, the presence of live cells indicating protection from virus cytopathic effect which is the neutralization of virus infectivity. The endpoint titer was calculated using a standard formula. A neutralizing antibody titer of 1:8 correlates with protection from disease and was used as the threshold of seropositivity. Each test was run in triplicate, with dilutions ranging from 1:8 to 1:1024. Each dried blood spot was tested for neutralizing antibodies against the Sabin 1, Sabin 2, and Sabin 3 oral vaccine strains.

### 2.3 Statistical analysis

**Fig. 2:**
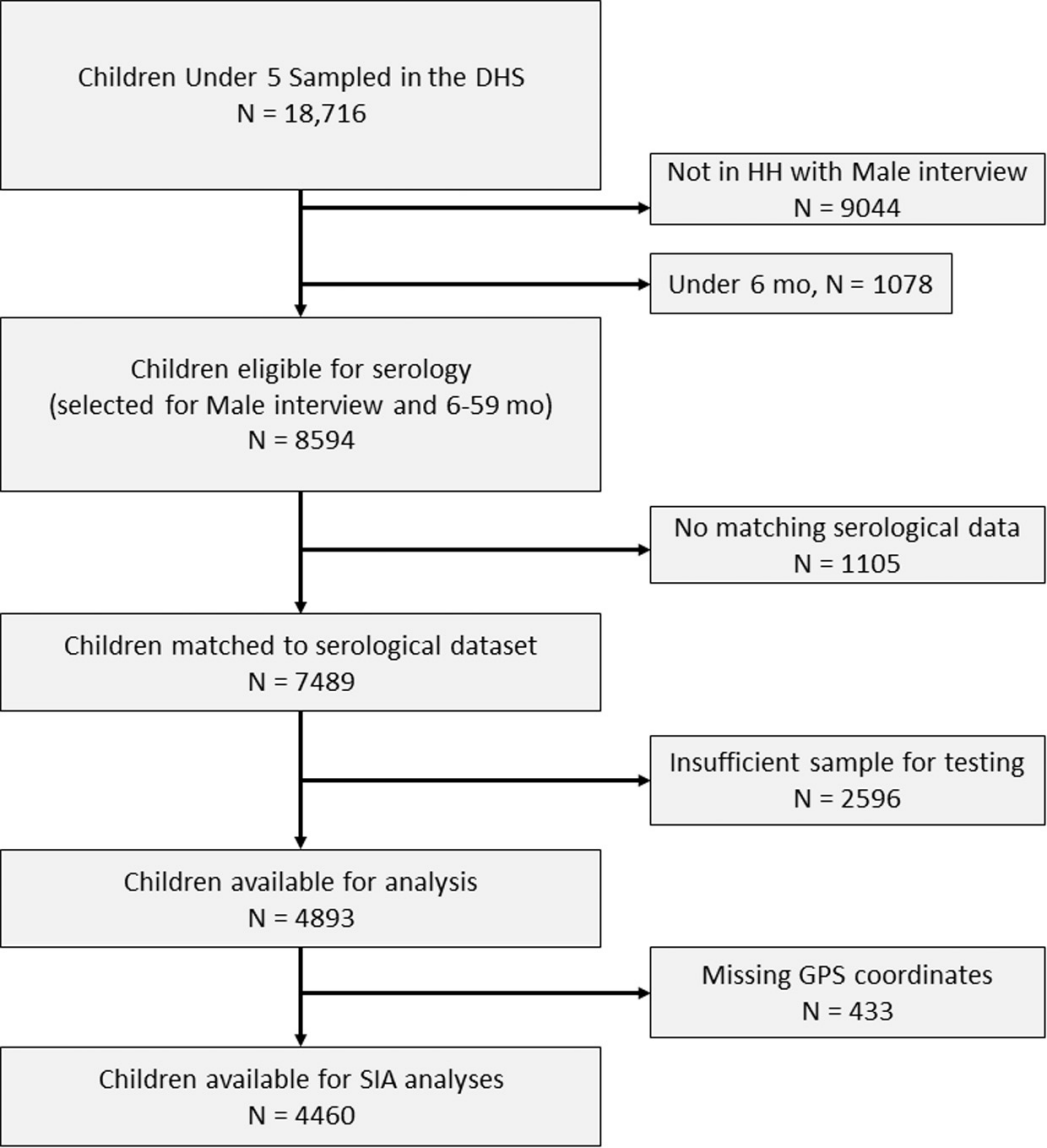
Sample size resulting from applying inclusion criteria and removing missing data.

Among 8594 eligible children, 1105 (13%) had no matching serological data, while a further 2596 had insufficient dried blood to perform the neutralizing assay (Fig. 2). This resulted in 4893 children available for analysis. The rate of missing data varied by province (30% in Equateur province, to 63% in Sud-Kivu), and by wealth index (40% in among the poorest and 49% among the richest). We accounted for missing data by raking the sampling weights so that each new province, age group, urban/rural group carried the same marginal weight as in the 8594 children eligible for analysis [21].

We augmented the information available in the DHS with the number of SIAs for which the child would have been eligible prior to the survey, noting the antigens present in the vaccine used. SIAs are organized by health district, which is not recorded in the DHS. We determined health districts from the DHS clusters’ GPS coordinates mapped onto health district boundaries used by the polio program. GPS coordinates were not available for 45 of the 536 clusters, and resulted in removal of 433 children from analyses involving SIAs. The SIAs for which a child would have been eligible was then determined by referencing the health district of the child at the time of blood draw against the SIA database maintained by WHO. This procedure assumes to some extent that the child lived in the same district during those SIAs. However, most SIAs target either the whole country or whole provinces, and thus the data should be robust to some amount of local travel.

When analyzing routine immunization status, we used diphtheria, tetanus and pertussis (DTP) vaccination as a proxy for vaccination with OPV, since they are given at the same time in the routine schedule and DTP is less likely to be confused with vaccine received through SIAs. This decision is consistent with previous analyses of poliovirus risk [6], [22]. We considered someone to have ‘partial’ routine immunization if they reported 1–2 doses of DTP, by recall or by vaccination card, and full immunization if they had 3 doses of DTP.

We estimated population immunity to each poliovirus serotype at the national level, by province, age group, wealth index, mothers’ education level, residence (rural or urban), household size, birth order, and routine immunization status. We also estimated population immunity to each serotype as a function of the number of SIAs for which a child was eligible. To test whether seroprevalence varied by a categorical variable (province, wealth index, education level, residence, routine immunization status), we compared an intercept-only binomial regression to a model that allows seroprevalence to vary with the characteristic, using the likelihood ratio test. For ordered numeric variables (age, household size, and birth order), this was done with a linear term. A p-value less than 0.05 was used to classify a result as statistically significant, which did not account for multiple testing.

To summarize differences in seroprevalence for differences in SIAs we used Poisson regression modified for binomial outcomes [23]. In this model, seroprevalence S as a function of the number of SIAs n took the form

(1) $$1-S(n)=\alpha \times {\left(1-\beta \right)}^{n}\text{.}$$

Here β is the proportional decrease in non-immune individuals 1-unit difference in n. This has the convenient interpretation as the seroconversion resulting from an additional SIA in an intention-to-treat analysis, averaged over differences in SIA eligibility in the population.

All analyses were performed in R version 3.2.3[24]. All analyses account for DHS survey design using the ‘survey’ package [21].

## 3 Results

**Fig. 3:**
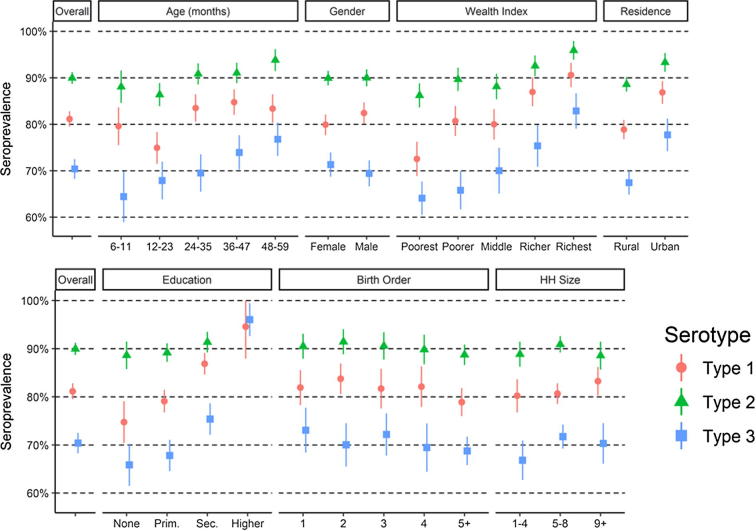
Seroprevalence by demographic characteristics.

Population immunity among 6–59-month olds to polio was 81% (CI: 79–83%), 90% (CI: 89–91%), and 70% (CI: 68–73%), for types 1, 2, and 3, respectively (Fig. 3 and Supplementary Table S1). Immunity also varied with basic demographic characteristics: for all three polio serotypes, immunity increased with age, wealth index, and mothers’ education, and tended to be higher in urban areas (p < 0.05). Immunity was not associated with the gender of the child, birth order, or household size. While immunity had a detectable increase with age, presumably due to SIAs and passively acquired immunity from contact with OPV recipients, the magnitude of the increase was relatively small; immunity to type 1 poliovirus was 79% among 6–11 month olds and 83% among 48–59 month olds. We observed relatively large differences in seroprevalence by wealth index; for type 1 poliovirus seroprevalence was 73% among children from the poorest households and 91% among the richest households.

**Fig. 4:**
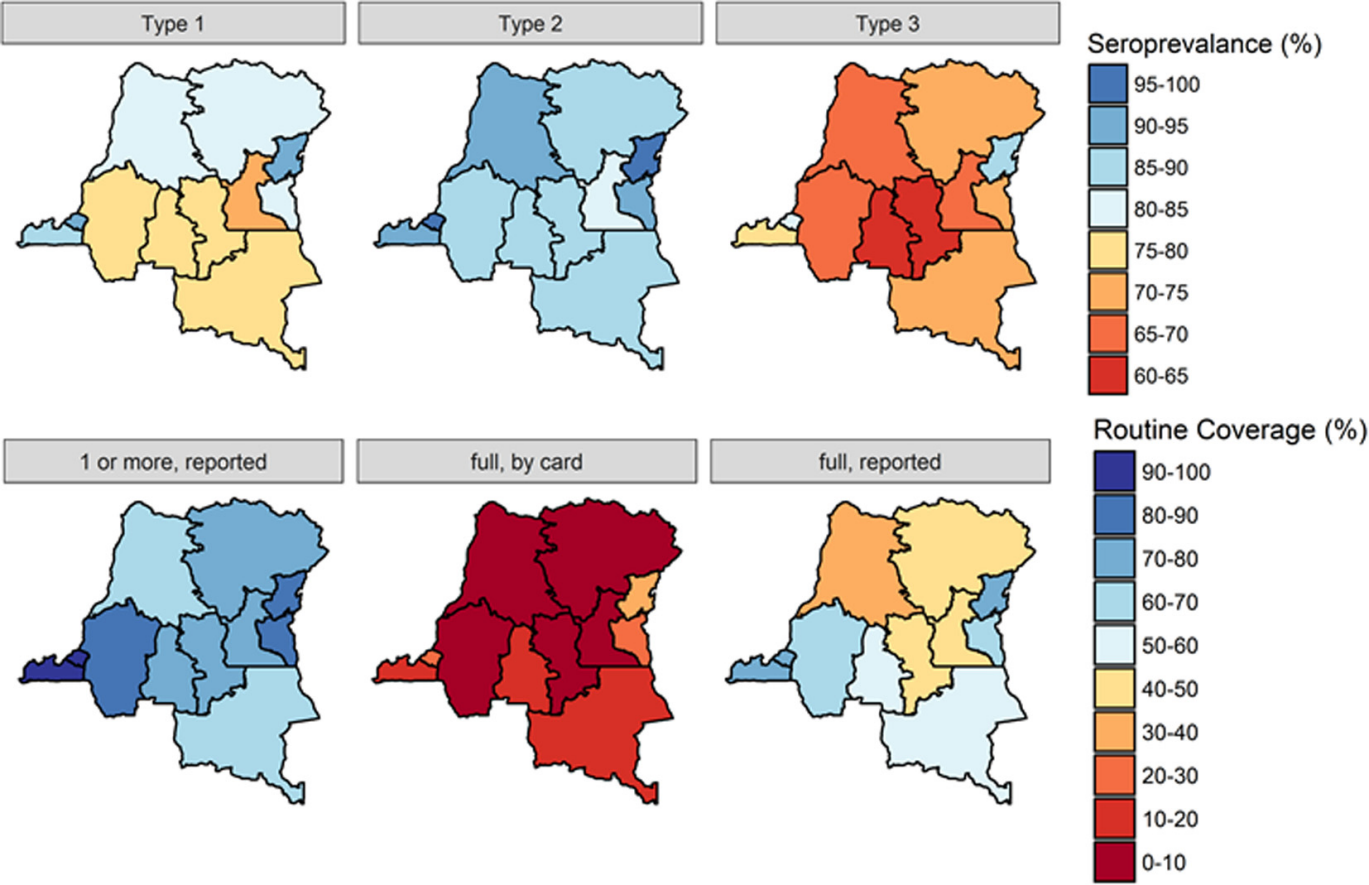
Seroprevalence and routine immunization coverage, by province.

We also found that population immunity to all three types of poliovirus varied by province (Fig. 4 and Supplementary Table S2). Immunity to each serotype was generally lower in central DRC, and higher in the east and west, and some regional variation reflects differences in wealth and residence. For instance, immunity to type 1 poliovirus is generally high in wealthy urbanized populations of Kinshasa. Not all provinces follow this trend, however. Sampled households in Nord-Kivu were neither particularly wealthy nor urbanized, but had high immunity to all three serotypes (Supplementary Fig. S1).

**Fig. 5:**
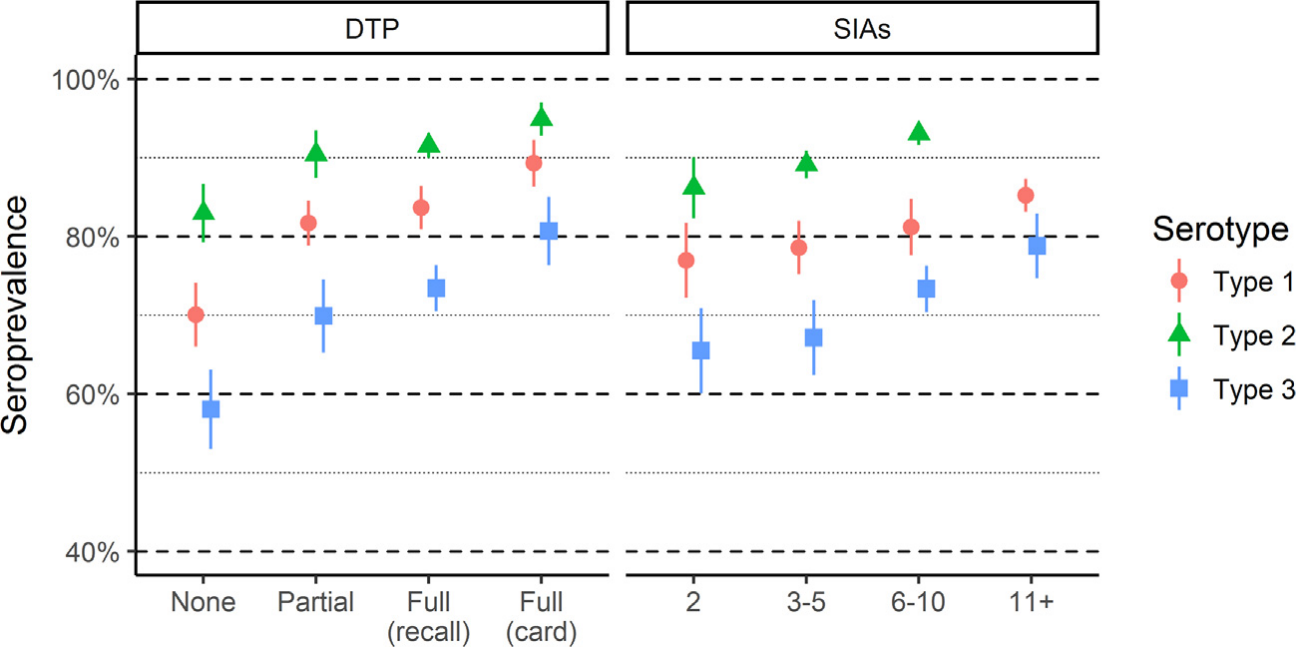
Seroprevalence by routine immunization status and SIA eligibility. Note, fewer SIAs with type-2 containing vaccine were conducted than for types 1 and 3. Thus, estimates for type 2 seroprevalence are not available for children who have experienced 11 or more SIAs.

We estimated the variation in seroprevalence by vaccination activity, assessing both routine immunization status and the number of SIAs for which a child would have been eligible (Fig. 5). Among all three polio serotypes, seroprevalence is higher among those who have received one or more doses of DTP compared to those who have not, whether by parental recall or vaccination card. Those with three documented doses of DTP have higher seroprevalence than those who report three doses based on parental recall, suggesting some inaccuracies in reported routine immunization status.

Immunity is higher among children who have experienced more SIAs. Note that all children in the study had experienced at least two tOPV SIAs. Among children who were eligible for additional SIAs, we estimate the (intention to treat) seroconversion to be 5.0% (CI: 4.2–5.8%), 16% (CI: 14–19%), and 5.5% (CI: 4.6–6.3%) per SIA for types 1, 2, and 3, averaged over relevant campaigns for each serotype.

**Fig. 6:**
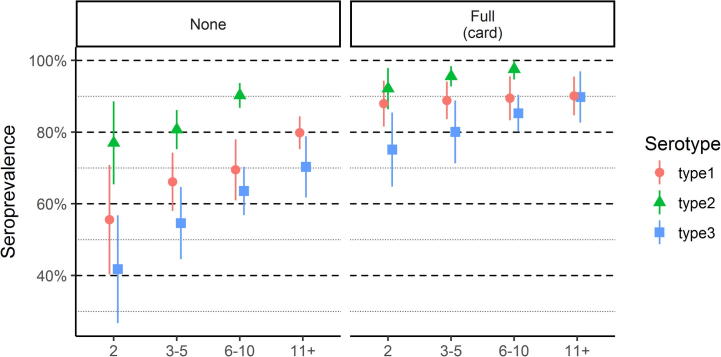
Seroprevalence by SIA eligibility, by stratified by routine immunization status. Left panel: those who report no DTP routine immunization. Right panel: those with documented full DTP immunization.

We stratified our analysis of SIA impact by routine immunization status (Fig. 6 and Supplementary Tables S3 and S4). Seroconversion rates did not vary significantly with routine immunization status (likelihood ratio test p-value > 0.05 for each serotype, comparing model with interaction term between SIA and DTP to a model with main-effects only). To isolate the impact of the first two tOPV SIAs, we examined seroprevalence among those who did not receive routine immunization and were eligible for two (tOPV) SIAs. Among this group, seroprevalance was 56% (40–71%), 77% (65–89%), and 42% (27–57%) for types 1, 2, and 3, respectively.

## 4 Discussion

Overall, our results indicate that immunity is moderately high for all three poliovirus serotypes (81%, 90%, and 70% for Polio Types 1, 2, and 3). Immunity was higher among the wealthy, educated and urban populations and, conversely, lower among children from poorer, less educated, or rural households. Variation in immunity among these demographic groups can be explained to some extent by routine immunization status and the number of immunization activities. For instance, 83% of those in the highest wealth quintile reported full DTP vaccination, compared to 44% of the poorest wealth quintile. However, there still remains substantial uncertainty about routine immunization coverage and the share of immunity that can be attributed to it, since only 19% of children had vaccination cards.

All children in the study were eligible for at least two tOPV SIAs; we found that immunity improved among older children who were eligible for additional SIAs. While the increase in immunity resulting from these SIAs was statistically significant, the increase in seroprevalence per-SIA was quite small, with seroconversion rates of 5.0%, 16%, and 5.5% for types 1, 2, and 3, respectively. These rates are much lower than is reported in clinical trial data, and also seem internally at odds with the high seroprevalence seen in recipients of routine immunization [8]. However, these are intention-to-treat estimates that only pertain to SIAs beyond the first two a child experienced. One explanation is that children who are missed in the first two SIAs are also likely to be missed in subsequent SIAs, and hence do not seroconvert. The relatively high seroconversion rate for type 2 OPV could then be attributed to its higher secondary spread to children missed by the SIA, in addition to higher vaccine efficacy. An alternative explanation is low OPV efficacy specific to SIA delivery, perhaps due to cold-chain management. However, seroprevalence among those who report no routine immunization and who were eligible for two tOPV SIAs was 56%, 77%, and 42% for types 1, 2, and 3, which suggests substantially higher seroconversion from those first two SIAs. Thus it seems likely that the vast majority of children who are not immunized after their first two SIAs will continue to be unimmunized following subsequent SIAs, at least for types 1 and 3. This in turn has implications for supplementary vaccination policy, where the GPEI may benefit more from increasing the quality of SIAs rather than their frequency, in addition to expanding routine immunization to less wealthy and less educated populations.

The design of the survey gives some insight into the geographic structure of immunity, and whether unimmunized children are found evenly throughout the clusters, or are concentrated in ones that are not reached by vaccinators. We estimate the intra-class correlation (ICC) to be 0.08, 0.05, and 0.09 for types 1, 2, and 3, respectively, after adjusting for survey strata. This moderately low ICC suggests that deficiencies in immunity are not explained by unvaccinated villages, but instead by dispersed unimmunized children within relatively well-vaccinated communities.

An important milestone in polio eradication occurred in April 2016 with the removal of type-2-containing OPV from use. Achieving high population immunity to type 2 poliovirus prior to removal of OPV2 is important to prevent emergence and transmission of VDPVs [25]. Our estimate of 90% immunity to type 2 poliovirus in a survey that occurred immediately after two national tOPV immunization campaigns was encouraging for its general success in the DRC. However, despite overall high immunity to type 2 poliovirus at the time of this survey, two separate cVDPV emergences were recently detected, with index cases identified in Maniema province in March 2017, and in central Katanga in April 2017 [2]. These areas had seroprevalence of 82% (74–93%) and 89% (86–92%), respectively, and were not significantly different from the country taken as a whole. Thus, it is possible that immunity declined in the intervening 3 years, or that cVDPVs emerged in pockets of low immunity that were not represented in the survey.

A follow-up survey was conducted in August 2016 in central Katanga, to study an area of frequent cVDPV emergence, as well as site of one of the 2017 outbreaks. Laboratory and data analyses from this study are ongoing will provide additional insight.

This study had several limitations. As a household-based survey, mobile or hard-to-reach populations may not be well-represented. The study was also cross-sectional, while some estimates, such as the intention-to-treat impact of an SIA, are longitudinal in nature and may be biased by changes in SIA or routine coverage over time. There was also a substantial proportion of missing serology data, which may have affected results if related to sero-status in a way which we did not account for.

## 5 Conclusions

Overall immunity was high at the time of the survey (2013–2014), though recent cVDPV emergences highlight the potential for pockets of unimmunized children. While routine immunization appears to confer high levels of immunity, the impact of repeated SIAs appears to be marginal. The GPEI would benefit from further serological study of high-risk populations and assessments of large-scale interventions such as SIAs.

## Figures and Tables

**Supplementary Fig. S1:**